# Irreversible electroporation ablation area enhanced by synergistic high- and low-voltage pulses

**DOI:** 10.1371/journal.pone.0173181

**Published:** 2017-03-02

**Authors:** Chenguo Yao, Yanpeng Lv, Shoulong Dong, Yajun Zhao, Hongmei Liu

**Affiliations:** The State Key Laboratory of Power Transmission Equipment & System Security and New Technology, School of Electrical Engineering, Chongqing University, Chongqing, China; Consiglio Nazionale delle Ricerche, ITALY

## Abstract

Irreversible electroporation (IRE) produced by a pulsed electric field can ablate tissue. In this study, we achieved an enhancement in ablation area by using a combination of short high-voltage pulses (HVPs) to create a large electroporated area and long low-voltage pulses (LVPs) to ablate the electroporated area. The experiments were conducted in potato tuber slices. Slices were ablated with an array of four pairs of parallel steel electrodes using one of the following four electric pulse protocols: HVP, LVP, synergistic HVP+LVP (SHLVP) or LVP+HVP. Our results showed that the SHLVPs more effectively necrotized tissue than either the HVPs or LVPs, even when the SHLVP dose was the same as or lower than the HVP or LVP doses. The HVP and LVP order mattered and only HVPs+LVPs (SHLVPs) treatments increased the size of the ablation zone because the HVPs created a large electroporated area that was more susceptible to the subsequent LVPs. Real-time temperature change monitoring confirmed that the tissue was non-thermally ablated by the electric pulses. Theoretical calculations of the synergistic effects of the SHLVPs on tissue ablation were performed. Our proposed SHLVP protocol provides options for tissue ablation and may be applied to optimize the current clinical IRE protocols.

## Introduction

Cell membrane permeability increases upon exposure to a high-voltage, pulsed electric field in a process known as electroporation [1]. After the action of the pulse, pores may persist in the membrane for a period of a few seconds to a few minutes without damaging the cell. This phenomenon, in which transient pores form in the membrane, is called reversible electroporation [2–4]. When stronger pulses act on the membrane, pores can become sufficiently large to cause irreversible damage to the membrane, leading to cell death. This phenomenon is called irreversible electroporation (IRE) [5–7].

In recent years, both types of electroporation have been applied with a wide variety of electromagnetic fields. Reversible electroporation is used for electrochemotherapy [8,9], gene electrotransfer [10,11], electrofusion [12,13] and nanoelectroporation [14–16]. IRE is used for bacterial inactivation [17], tumor ablation [18–20], food processing and environmental management [21].

In the laboratory, electroporation experiments have been performed using animal models [22–24] as well as fruit and vegetable tissue models; in particular, potato slices have been used to evaluate electric field distributions, impedance changes and tissue damage after IRE [25–28]. For potato slices, damage occurs through oxidation via the release of intracellular polyphenol oxidases, and such damage manifests as dark areas caused by membrane rupture. In addition, potato tissue has been shown to reflect the effects of electroporation observed in vivo, and lesion boundaries can be easily defined by a marked darkening of the treated areas [29]. Therefore, these dark areas are suitable for representing the effects of IRE treatment.

The development of electroporation is dependent on the electric pulse protocol [30–40]. Christopher B. Arena et al. demonstrated greatly improved outcomes in heterogeneous tissues using high-frequency bipolar pulses [37]. Richard Heller and Mojca Pavlin found that a combination of short high-voltage pulses and long low-voltage pulses could enhance the efficiency of gene electrotransfer [38,39]. In addition, IRE has been very successful in the treatment of tumors [40]. The traditional pulse used for IRE ranges from tens of volts to tens of kilovolts per centimeter, with pulse widths ranging from nanoseconds to milliseconds [35,36]. The strength of the electric field and the width of the pulse are key predictors of the degree of tissue ablation. Optimization of the pulse electrode configuration is necessary to ensure that there is full tumor coverage while avoiding damage to normal tissue [40]. In general, a shorter pulse width requires a stronger electric field for tissue ablation. However, in a strong electric field, increasing the pulse width may cause injury to a human subject.

In this study, we propose a protocol that combines short high-voltage pulses (HVPs) and long low-voltage pulses (LVPs) to enhance IRE for tissue ablation. Electroporation occurs easily with the application of HVPs, producing large electroporated regions in tissue; however, IRE occurs only near the electrodes, and long HVPs will induce a thermal effect [41,42]. LVPs can also produce IRE, but it is more difficult to achieve electroporation with weaker electric fields; thus, the ablation area would remain small. However, the combination of HVPs and LVPs will synergistically enhance the area of tissue ablation. With stronger electric fields, HVPs create larger electroporated areas that are more susceptible to subsequent passes of LVPs. This combination of HVPs and LVPs for tissue ablation is called the synergistic high- and low-voltage pulse (SHLVP) protocol.

To validate our approach, we applied different electric pulse protocols to potato tissue slices, including HVP, LVP, SHLVP (HVP+LVP) and LVP+HVP protocols. The results demonstrated that the SHLVP protocol produced the ablation area more effectively than either the HVP or LVP protocol, even when the SHLVPs were applied with equal or reduced electric pulse doses compared with those of the HVPs or LVPs applied alone. In contrast, the LVP+HVP protocol did not result in enhanced tissue ablation. In addition, we showed that the tissue was non-thermally ablated by the protocols evaluated in this study. In the discussion, theoretical considerations underlying the effect of SHLVPs for tissue ablation are evaluated. Our method may be applied to optimize current IRE protocols and enhance the outcome of ablation therapies.

## Methods

**Fig 1:**
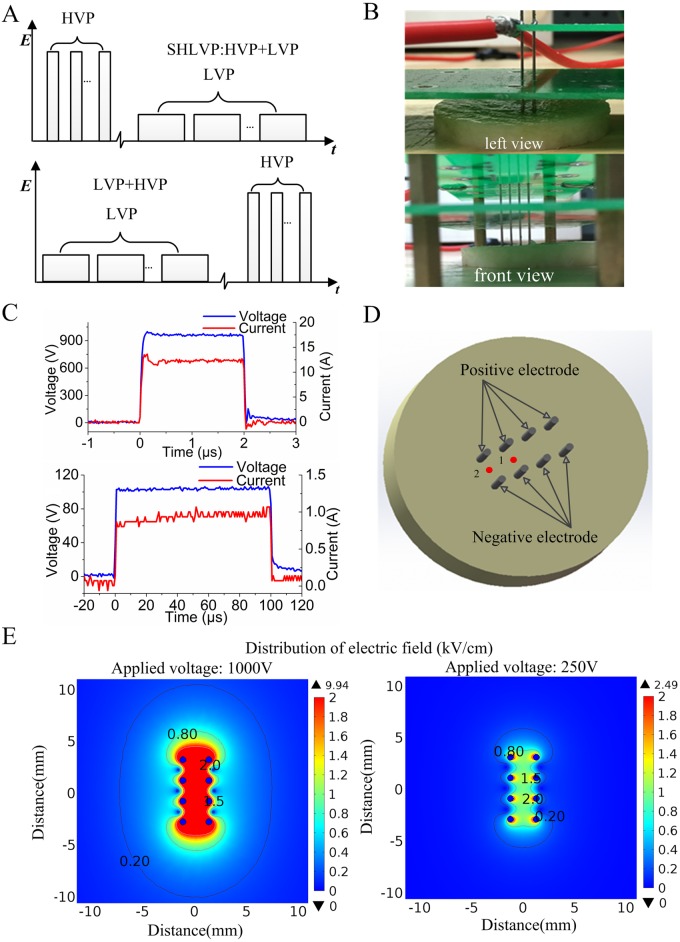
A: Schematic of the protocols. B: Images of the construction of the experimental electrodes; the left view is shown at the top, and the front view is shown at the bottom. C: Waveforms of 4 kV/cm, 2 μs (top), and 0.4 kV/cm, 100 μs (bottom). D: Temperature measurement points (‘1’ and ‘2’) in the potato tissue. E: The distribution of electric field for 1000 V (left) and 250 V (right) applied.

Our experimental setup was based on several pulse protocols that were applied to potato tuber slices. The SHLVPs were achieved by a pulse generator with dual power charging through a MOSFET circuit. To encourage heat dissipation and prevent the potato from blackening due to overheating, we used cylindrical slices with a diameter of 50 mm and a height of 6 mm. Potato slices were ablated with an array of four pairs of parallel steel electrodes, each 40 mm long and 0.6 mm in diameter. The electrodes were inserted 6 mm deep into the slices with 2.5 mm between the positive and negative electrodes and 2 mm between electrodes of the same polarity. Fig 1A shows a schematic of the protocols, and Fig 1B and 1D show the construction of the experimental electrodes. The electric field intensity from eight needle electrodes has the nonlinear, inhomogeneous distribution, which varies spatially, thus highly spatially inconstant. Therefore, the voltage-to-distance (kV/cm) ratio is provided as a general reference metric for pulsed electric field strength based on needle separation and voltage. For instance, if the pulse voltage is 1000 V, the pulsed electric field (the voltage-to-distance (kV/cm) ratio is a general reference metric) is 4 kV/cm. Fig 1E shows the electric field distribution for the applied voltage of 1000 V (left) and 250 V (right).

Four electric pulse protocols (HVP, LVP, SHLVP [HVP+LVP] and LVP+HVP) were applied to the potato slices to demonstrate the synergistic effect of the HVP and LVP components of the SHLVP protocol. The electrical dose was used to facilitate a comparison, as described by the following equation:

$$Dose=\sum_{n=1}^{N}E_{{}^{n}}^{2}\times T_{n}[kV^{2}\mu s/cm^{2}]$$ (1)

where *E*_*n*_ is the electric field of the *n*th pulse, *T*_*n*_ is the duration of the *n*th pulse, and *N* is the total number of pulses. The HVPs had an electric field strength of either 3 kV/cm or 4 kV/cm and consisted of either 20 or 80 pulses with a pulse width of 2 μs at 1 Hz; the width calculation is shown in the discussion. The LVPs had an electric field strength ranging from 0.4–1 kV/cm and a pulse width of 100 μs, and either 60 or 80 pulses were applied at 1 Hz. The time interval between the individual HVP and LVP protocols was 1 s. The LVP protocol that included 80 LVPs applied alone was denoted as LVP1. The LVP protocol that included 60 LVPs applied alone was identified as LVP2. The HVP protocol that included 80 HVPs applied alone (4 kV/cm, 2 μs) was denoted as HVP1. To demonstrate the enhancement of the ablation area by SHLVPs (HVPs+LVPs), experiments involving tissue ablation by different pulsed electric protocols were divided into five sections.

On the premise that the SHLVPs (HVPs+LVPs) had a similar dose as the LVP1 (only LVPs), an experiment was conducted to illuminate whether the SHLVPs would result in a larger ablation area. The schemes are listed in Table 1, in which each group had the similar dose.

**Table 1:** Schemes for pulsation protocols with the similar dose.

	HVP			LVP			Total dose
	E (kV/cm)	T (μs)	N	E (kV/cm)	T (μs)	N	(kV^2^μs/cm^2^)
**LVP1**				0.4	100	80	1280
**SHLVP-a**	3	2	20	0.4	100	60	1320
**SHLVP-b**	4	1	20	0.4	100	60	1280
**LVP1**				0.6	100	80	2880
**SHLVP-a**	3	4	20	0.6	100	60	2880
**SHLVP-b**	4	2	20	0.6	100	60	2800
**LVP1**				0.8	100	80	5120
**SHLVP-a**	3	7	20	0.8	100	60	5100
**SHLVP-b**	4	4	20	0.8	100	60	5120
**LVP1**				1	100	80	8000
**SHLVP-a**	3	11	20	1	100	60	7980
**SHLVP-b**	4	6	20	1	100	60	7920

Regarding the influence of SHLVP sequence, when SHLVPs (HVPs+LVPs) were applied to the tissue, the LVPs were applied after the HVPs; however, for reverse SHLVPs (LVPs+HVPs), the sequence was the reverse. The schemes are listed in Table 2.

**Table 2:** Schemes for SHLVPs with different sequences.

	HVP			LVP		
	E (kV/cm)	T (μs)	N	E (kV/cm)	T (μs)	N
**SHLVPs (HVPs+LVPs)**	4	2	20	0.4	100	60
**Reverse SHLVPs (LVPs+HVPs)**	4	2	20	0.4	100	60
**SHLVPs (HVPs+LVPs)**	4	2	20	0.6	100	60
**Reverse SHLVPs (LVPs+HVPs)**	4	2	20	0.6	100	60
**SHLVPs (HVPs+LVPs)**	4	2	20	0.8	100	60
**Reverse SHLVPs (LVPs+HVPs)**	4	2	20	0.8	100	60
**SHLVPs (HVPs+LVPs)**	4	2	20	1	100	60
**Reverse SHLVPs (LVPs+HVPs)**	4	2	20	1	100	60

On the premise that the dose of the SHLVPs (HVPs+LVPs) was less than that for the LVP1 (LVPs applied alone), the experiments were designed to indicate whether the ablation area of SHLVPs was still larger than that of LVP1. For example, under the same LVP electric field (0.8 kV/cm) for both SHLVPs (HVPs+LVPs) and LVP1 (LVPs applied alone), the dose of SHLVPs (both SHLVP-3 kV and SHLVP-4 kV) was less than that of LVP1. The schemes are listed in Table 3.

**Table 3:** Schemes for pulsation protocols with different doses.

	HVP			LVP			Total dose
	E (kV/cm)	T (μs)	N	E (kV/cm)	T (μs)	N	(kV^2^μs/cm^2^)
**LVP2**				0.8	100	60	3840
**LVP1**				0.8	100	80	5120
**SHLVP-3 kV**	3	2	20	0.8	100	60	4200
**SHLVP-4 kV**	4	2	20	0.8	100	60	4480
**LVP1**				1	100	80	8000
**SHLVP-4 kV**	4	2	20	1	100	60	6640

Under the same parameters of HVPs, the ablation areas of SHLVPs (HVPs+LVPs) were explored with the applied LVP electric field of 0.4 to 1 kV/cm. The ablation areas of LVP2 (only LVPs) were also studied. The schemes are listed in Table 4.

**Table 4:** Schemes for pulsation protocols with different LVP electric fields.

	HVP			LVP		
	E (kV/cm)	T (μs)	N	E (kV/cm)	T (μs)	N
**SHLVP-4 kV**	4	2	20	0.4	100	60
**SHLVP-4 kV**	4	2	20	0.6	100	60
**SHLVP-4 kV**	4	2	20	0.8	100	60
**SHLVP-4 kV**	4	2	20	1	100	60
**LVP2**				0.4	100	60
**LVP2**				0.6	100	60
**LVP2**				0.8	100	60
**LVP2**				1	100	60

For the same pulse number, the ablation areas from applying SHLVPs (20 HVPs+60 LVPs) were compared to HVP1 (80 HVPs applied alone) and LVP1 (80 LVPs applied alone). The protocols are listed in Table 5.

**Table 5:** Schemes for pulsation protocols with the same pulse number.

	HVP			LVP			Total pulse number
	E (kV/cm)	T (μs)	N	E (kV/cm)	T (μs)	N	
**HVP1**	4	2	80				80
**LVP1**				0.6	100	80	80
**SHLVP-4 kV**	4	2	20	0.6	100	60	80

The voltage and current were monitored in real time with a LeCroy oscilloscope (WavePro760Zi-A, bandwidth: 6 GHz, USA) and a 411 Pearson current probe (Pearson Electronics Inc., Palo Alto, USA); the voltage and current waveforms were synchronous (Fig 1C). The energy injected through electric pulsation is given by the following equation:

$$W=\int_{0}^{t}UIdt$$ (2)

where *U* is the voltage of the pulse, and *I* is the current of the pulse.

The lesions of the ablated potato slices were imaged (EOS 1200D, Canon) for analysis 12 hours after the experiment. The surfaces of the slices were darkened by oxidization, which could lead to an overestimation of the ablation area. Therefore, the slices were cut down the middle in the direction of the electric field to access a region unaffected by air contact. ImageJ software and MATLAB 2014 software were used to calculate the dark areas (ablation area) in the photographs. Real-time temperature changes were recorded using a fiber optic sensor (FOT Lab Kit, LumaSense Technologies, USA). The temperature measurement points are shown in Fig 1D (labeled ‘1’ and ‘2’). All the data were statistically analyzed by performing ANOVA using Microsoft Excel. The data are presented as the means ± standard deviation (SD) for *n* independent experiments. The experiments were repeated at least 3 times for each protocol.

## Results

### Combining Short High- with Long Low- Voltage Pulses (SHLVPs) increase IRE ablation efficiency

**Fig 2:**
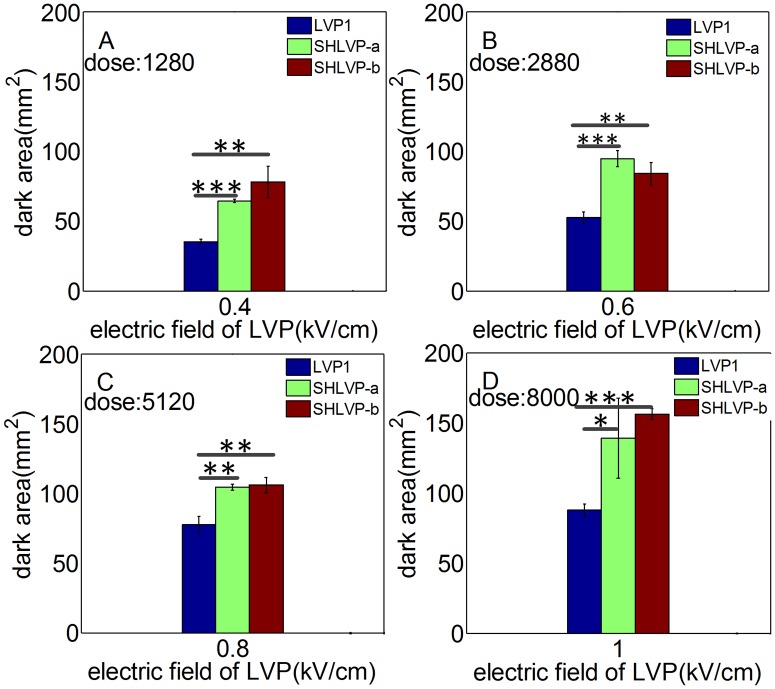
For the similar dose, the mean ablated region (dark area) resulting from SHLVP is larger than that of LVP1. Note that 80 LVPs were used in the LVP1 protocol. The duration of the HVPs was adjusted to yield approximately the same dose for the SHLVP and LVP1 protocols. Therefore, the doses shown in Fig 2A, B, C and D produced in the three groups (LVP1, SHLVP-a and SHLVP-b) were the similar dose. All detailed protocol parameters are shown in Table 1. Data are shown as the average ± SD; (*, p<0.05), (**, p<0.01), and (***, p<0.001).

We performed an experiment to determine whether the ablation area could be enhanced by SHLVPs compared with LVP1 applied at similar electrical doses. The detailed parameters used are listed in Table 1 (in Method). The LVP protocol included 80 LVPs and was denoted as LVP1. The width of the HVPs was adjusted to yield protocols SHLVP-a and SHLVP-b (both 20 HVPs+60 LVPs), which delivered the similar dose as that of the LVP1 protocol while keeping the electric field of the LVPs constant. The results of ablation area by different protocols are presented in Fig 2. The synergistic effect of the SHLVPs resulted in strong ablation at the similar dose. For example, in Fig 2A, at the similar dose (1280 kV^2^μs/cm^2^) and the same LVP electric field (0.4 kV/cm), the SHLVP-a and SHLVP-b ablation areas were larger than that of LVP1, in which only 80 LVPs were applied. When the electric field of the LVPs was adjusted to 0.6 kV/cm (Fig 2B), the dose increased (2880 kV^2^μs/cm^2^). However, when the same dose and LVP electric field were applied, the SHLVP-a and SHLVP-b ablation areas remained larger than that of LVP1, even when the electric field of the LVPs was as high as 1 kV/cm (Fig 2D). Therefore, the electrical dose did not affect the synergistic enhancement of the ablation area. Notably, as shown in Fig 2C, SHLVP-b (4.25 J) and LVP1 (4.76 J) had similar levels of injected energy. However, the ablation area of SHLVP-b (106.22 mm^2^) was greater than that of LVP1 (77.42 mm^2^), suggesting that the synergistic effect of SHLVPs may be independent of the injected energy.

### Sequence of combining Short High- with Long Low- Voltage Pulses (SHLVPs) influences ablation area

**Fig 3:**
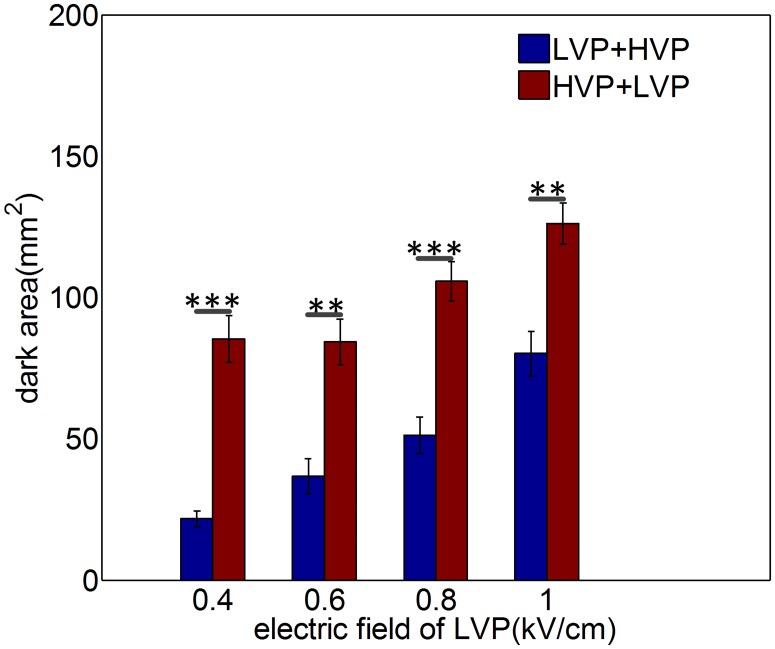
Mean ablation areas are influenced by sequence of SHLVPs (HVPs+LVPs). The parameters of the HVPs were 4 kV/cm, 2 μs and 20 pulses; the parameters of the LVPs were 0.4–1 kV/cm, 100 μs and 60 pulses. Data are shown as the average ± SD; (**, p<0.01) and (***, p<0.001). Detailed protocol parameters are shown in Table 2.

SHLVPs (HVPs+LVPs) had a positive effect on ablation area enhancement. However, changing the sequence of the HVP and LVP treatments did not have a markedly different effect. The detailed parameters used are listed in Table 2 (in Method). Fig 3 shows the ablation areas observed for two pulse protocols, i.e., HVP+LVP and LVP+HVP, as the electric field of the LVPs increased from 0.4 to 1 kV/cm. The HVP protocol used 4 kV/cm and 2 μs. The HVP+LVP protocol generated a larger ablation area than did the LVP+HVP protocol. The function of the HVPs is to create a larger electroporated area, thus increasing the susceptibility of the area to the LVPs. The larger ablation area resulting from SHLVPs lends support to this point. In contrast, the reverse order (LVPs+HVPs) did not achieve the same effect; even if an electroporated area was created by the HVPs, there were no subsequent LVPs to ablate the tissue. Thus, the LVP+HVP protocol ablated tissues less effectively than the SHLVP protocol.

### SHLVPs increase IRE ablation efficiency at lower doses compared with LVP1

**Fig 4:**
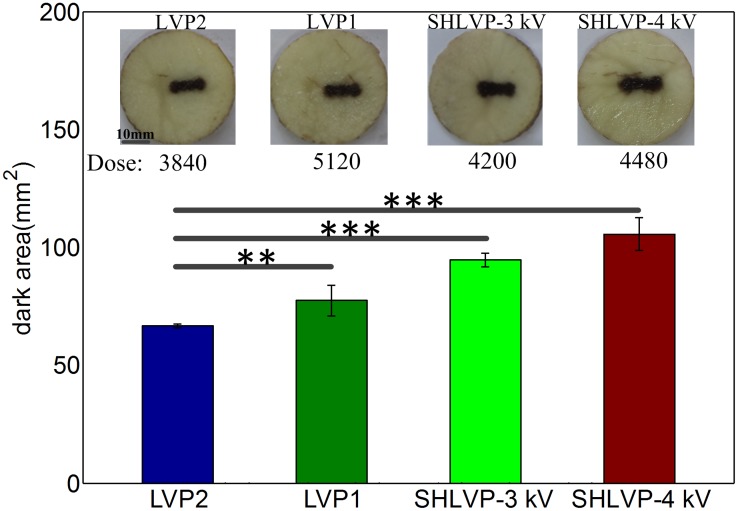
For a lower dose, applying SHLVPs produces a larger ablation area than that of LVP1. The parameters were as follows: LVP2: 0.8 kV/cm, 100 μs, 60 pulses; LVP1: 0.8 kV/cm, 100 μs, 80 pulses; SHLVP-3 kV: 3 kV/cm, 2 μs, 20 pulses+0.8 kV/cm, 100 μs, 60 pulses; SHLVP-4 kV: 4 kV/cm, 2 μs, 20 pulses+0.8 kV/cm, 100 μs, 60 pulses. Data are shown as the average ± SD; (**, p<0.01) and (***, p<0.001). Detailed protocol parameters are shown in Table 3.

The ablated area resulting from SHLVPs was larger than that of LVP1 delivered at an equivalent dose. However, compared with applying LVP1, applying SHLVPs at a lower dose could still generate a larger ablated area. The LVP protocol that included 60 LVPs was denoted as LVP2. The LVP protocol that included 80 LVPs was denoted as LVP1. The detailed pulse protocols are listed in Table 3 (in Method). The results are shown in Fig 4. When LVP2 (only 60 LVPs) was applied at a dose of 3840 kV^2^μs/cm^2^, the ablated area was 66.69 mm^2^. When LVP1 (only 80 LVPs) was applied, the dose increased to 5120 kV^2^μs/cm^2^, which was 25% greater than that delivered by LVP2 (only 60 LVPs); however, the ablated area (77.41 mm^2^) was only 16.09% greater than that resulting from LVP2. When 20 HVPs with the electric field of 3 kV/cm were applied and were followed by 60 LVPs (SHLVP-3 kV), the dose of SHLVP-3 kV (4200 kV^2^μs/cm^2^) increased by only 9.03% compared to that of LVP2 (only 60 LVPs). However, the ablation area was 94.72 mm^2^, 42.03% greater than that resulting from LVP2. Therefore, under the same parameter of LVP, applying SHLVP-3 kV could enhance the ablation area with a lower dose than that of LVP1. This enhancement of the ablated area was more pronounced using the SHLVP-4 kV (105.65 mm^2^) protocol, in which the HVPs were stronger (4 kV/cm). SHLVP-4 kV (HVPs+LVPs: 4 kV/cm +0.8 kV/cm) was applied to tissue at a dose of 4480 kV^2^μs/cm^2^. The dose of SHLVP-4 kV was 640 kV^2^μs/cm^2^ lower than the dose of LVP1 (LVPs only: 0.8 kV/cm). In contrast, the ablation area resulting from SHLVP-4 kV was 105.65 mm^2^, which was 28.24 mm^2^ greater than that of LVP1 (LVPs only: 0.8 kV/cm). Our analysis revealed that the dose of LVP1 was higher than that of SHLVPs; however, the ablation area of LVP1 was lower than that of SHLVPs. Therefore, applying SHLVPs generated a larger ablation region than that of LVP1 even when the dose of SHLVPs was lower than that of LVP1.

**Fig 5:**
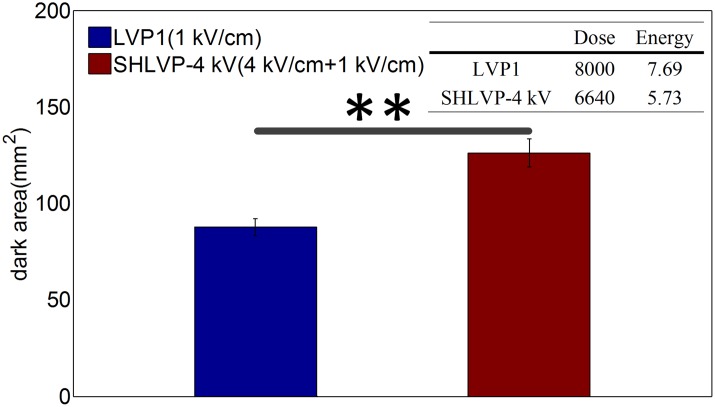
For both a lower dose and applied energy, the mean ablated region by SHLVP is still larger than that of LVP1. Parameters: LVP1 (1 kV/cm, 100 μs, 80 pulses) and SHLVP-4 kV (4 kV/cm, 2 μs, 20 pulses+1 kV/cm, 100 μs, 60 pulses). Data are shown as the average ± SD; (**, p<0.01). Detailed protocol parameters are shown in Table 3.

The high efficiency of the SHLVP-4 kV protocol could also be observed at lower doses and lower applied energy levels. The detailed pulse protocols are listed in Table 3 (in Method). As shown in Fig 5, both the dose and applied energy of SHLVP-4 kV (4 kV/cm+1 kV/cm, dose: 6640 kV^2^μs/cm^2^, injected energy: 5.73 J) were lower than those of LVP1 (1 kV/cm, dose: 8000 kV^2^μs/cm^2^, injected energy: 7.69 J), but a larger ablation area (126.07 mm^2^) was still observed for SHLVP-4 kV than for LVP1 (87.59 mm^2^). This result demonstrates that the synergistic effect of SHLVPs created a larger ablation area than that of LVP1 even when the dose and applied energy of SHLVP-4 kV were lower than those of LVP1.

### IRE ablation efficiency is enhanced by increased LVP electric field component of SHLVPs

**Fig 6:**
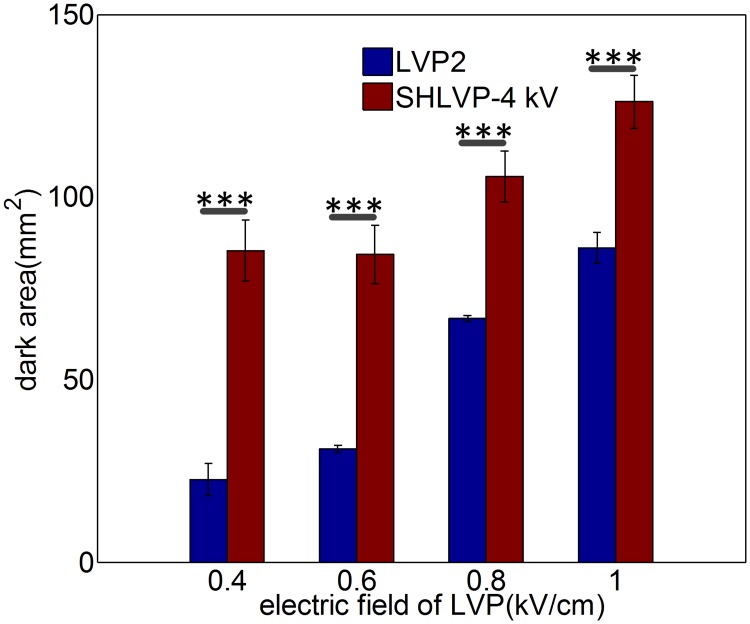
Increased electric field strength of the LVP component of SHLVPs augments the ablation area. The parameters were as follows: LVP2: 0.4–1 kV/cm, 100 μs, 60 pulses; SHLVP-4 kV, including HVPs (4 kV/cm, 2 μs, 20 pulses) and LVPs (0.4–1 kV/cm, 100 μs, 60 pulses). Data are shown as the average ± SD; (***, p<0.001). Detailed protocol parameters are shown in Table 4.

Under the same HVPs parameters, the increase in ablation area positively correlated with the electric field strength of the LVPs component when SHLVPs (HVPs+LVPs) were applied. The detailed pulse protocols are shown in Table 4 (in Method). The results are shown in Fig 6. In addition, on the premise of the same HVP parameters (20 HVPs), the ablation area resulting from LVP2 (60 LVPs alone) was enhanced one- to four-fold, depending on the LVP electric field strength (0.4 to 1 kV/cm) when 20 HVPs were applied prior to 60 LVPs (SHLVP-4 kV).

### SHLVPs increase IRE ablation efficiency with the same pulse number as HVP1 and LVP1

**Fig 7:**
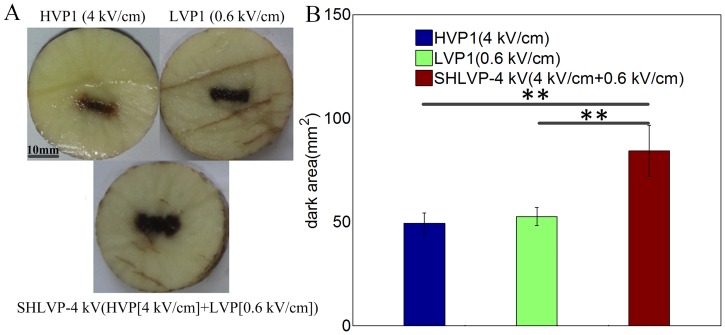
Comparison of ablation regions with the same pulse number subjected to HVP1, LVP1 and SHLVP-4 kV (HVP+LVP) pulsation. A: Images of the ablated regions. B: The mean ablated regions (dark areas) depicted in A. Parameters: HVP1 (4 kV/cm, 2 μs, 80 pulses); LVP1 (0.6 kV/cm, 100 μs, 80 pulses); and SHLVP-4 kV (4 kV/cm, 2 μs, 20 pulses+0.6 kV/cm, 100 μs, 60 pulses). Data are shown as the average ± SD (**, p<0.01). Detailed protocol parameters are shown in Table 5.

To further investigate the high efficiency of SHLVPs for tissue ablation under the same pulse number, the HVP1 (80 HVPs alone) protocol was applied to the potato slices. The detailed pulse protocols are listed in Table 5 (in Method). The HVP1 protocol (80 HVPs alone) had the same number of pulses as the LVP1 (80 LVPs alone) and SHLVP-4 kV (20 HVPs+60 LVPs) protocols. Fig 7B shows the ablation areas obtained using the three pulsation protocols. When HVP1 (80 HVPs alone) was applied, the ablation area obtained was 49.28 mm^2^. When LVP1 (80 LVPs alone) was applied, an ablation area of 52.58 mm^2^ was obtained, which was similar to that resulting from HVP1. However, the ablation area resulting from SHLVP-4 kV (20 HVPs+60 LVPs) was 84.22 mm^2^, which was increased by approximately 60% relative to that of LVP1 (80 LVPs alone) or HVP1 (80 HVPs alone). Therefore, on the condition that the SHLVP protocol had the same pulse number as HVP1 and LVP1, the ablation area when either HVP1 or LVP1 applied was lower than that of SHLVP. In addition, the darkness of the ablation area varied with the three protocols. As shown in Fig 7A, the area ablated by HVP1 was lighter in color than those of ablated by LVP1 and SHLVP-4 kV. Because the pulse width of the HVPs was shorter, IRE was more difficult to achieve. Furthermore, the energy applied by HVP1 was lower than that applied by LVP1 and SHLVP-4 kV. These two factors may explain the lighter color of the HVP1 ablation area.

### Non-thermal ablation with the three pulsation protocols

**Fig 8:**
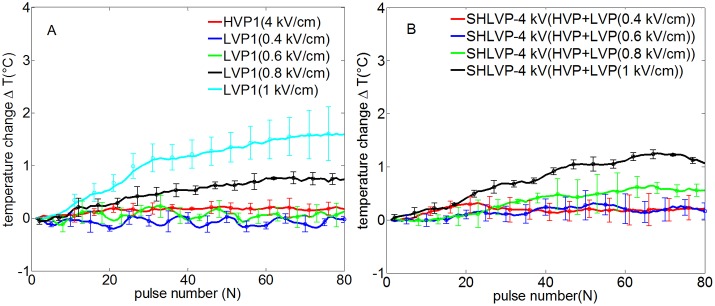
Real-time temperature changes at point 1 as a function of the number of HVPs, LVPs (A) and SHLVPs (B). HVP1: 4 kV/cm, 2 μs, 80 pulses; LVP1: 0.4–1 kV/cm, 100 μs, 80 pulses; SHLVP-4 kV: HVPs (4 kV/cm, 2 μs, 20 pulses)+LVPs (0.4–1 kV/cm, 100 μs, 80 pulses). Note that the inclusion of 80 error data points in the figure would make it difficult to differentiate different protocols. Therefore, partial error data are provided in Fig 8. Data are shown as the average ± SD.

**Fig 9:**
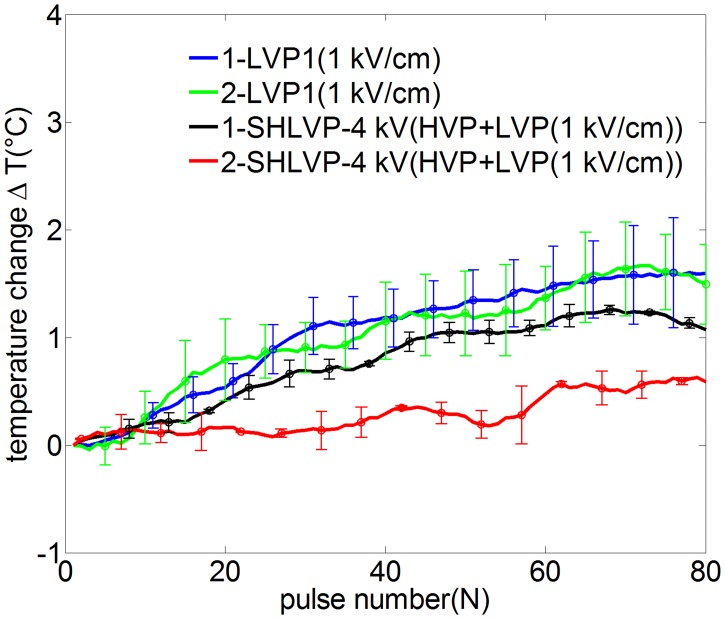
Real-time temperature changes at points 1 and 2 as a function of the number of pulsations in LVP1 and SHLVP-4 kV. LVP1: 1 kV/cm, 100 μs, 80 pulses; SHLVP-4 kV: HVPs (4 kV/cm, 2 μs, 20 pulses)+LVPs (1 kV/cm, 100 μs, 80 pulses). Note that the inclusion of 80 error data points in the figure would make it difficult to differentiate different protocols. Therefore, partial error data are provided in Fig 9. Data are shown as the average ± SD.

As non-thermal ablation is an important consideration for the HVP, LVP and SHLVP protocols, the temperature should be kept within given limits. The points used for real-time temperature monitoring are shown in Fig 1D. At point 1 (Fig 8A and 8B), as the tissue was exposed to more pulses, the temperature increased for LVP1 and SHLVP-4 kV only when the electric field of the LVPs was greater than 0.6 kV/cm. There was a positive correlation between the rise in temperature and the strength of the LVP electric field. The greatest temperature changes were 1.6°C for LVP1 and 1.1°C for SHLVP-4 kV. When HVP1 was applied alone, no thermal change occurred. There were no significant differences between the temperature changes at points 1 and 2 for LVP1 and SHLVP-4 kV (Fig 9). Therefore, these findings confirmed that the tissues were non-thermally ablated by the electric pulses.

## Discussion

### Theoretical analysis of the synergistic effects of SHLVPs

**Fig 10:**
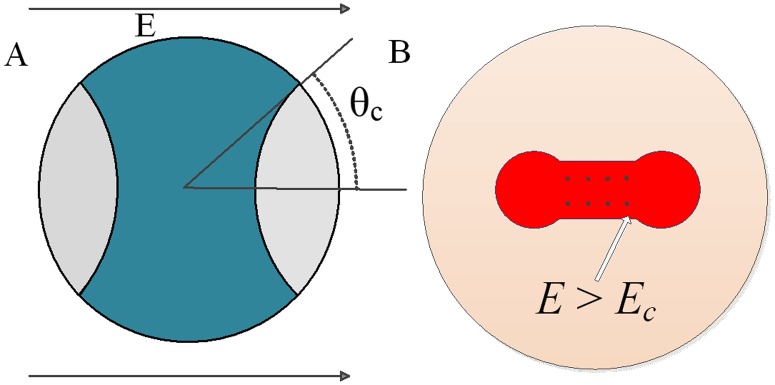
A: Model of a spherical electroporated cell exposed to an external electric field *E*. B: Model of potato tissue exposed to an external electric field *E*; the red section represents the electroporated area, where *E*>*E*_*c*_ when HVPs are applied.

HVPs can create an environment in which electroporated tissues are easily necrotized. When a spherical cell is exposed to an external electric field *E*, a transmembrane voltage *U*_*m*_ is induced:

$$U_{m}=\frac{3}{2}Er\text{cos}\theta (1-e^{-t/\tau }),\tau =rC(\frac{1}{2s_{e}}+\frac{1}{s_{i}})$$ (3)

where *θ* is the angle between the radial direction of a point on the membrane and the direction of the electric field, *r* is the cell radius (10 μm), *E* is the external electric field, *s*_*i*_ is the intracellular conductivity (0.455 S/m), *s*_*e*_ is the extracellular conductivity (0.03 S/m), *C* is the surface capacitance of the membrane (0.01 F/m^2^), and *τ* is the charge constant (1.89 μs). Electroporation and increased permeability will occur in the region of the cell membrane where *U*_*m*_ exceeds the critical value *U*_*c*_. The critical electric field *E*_*c*_ is defined (*θ* = 0, Fig 10A) as the following:

$$E_{c}=U_{c}/1.5r$$ (4)

From the above equations, the total electroporated area of the cell membrane that exceeds the critical transmembrane voltage *U*_*c*_ is given by the equation below [38]:

$$S_{c}=S(1-\frac{E_{c}}{E})$$ (5)

where *S* is the total surface area of the cell membrane. Eq 5 shows the positive proportional relationship between the electroporated area of the membrane and the external electric field. In tissues that contain massive cells, the electroporated area can be obtained by the following equation [43]:

$$S_{t}=\iint_{\Sigma }dSE>E_{c}$$ (6)

Ʃ is the tissue area where the electric field *E* is above the critical electric field *E*_*c*_. Eq 6 is a surface integral over the region in which the electric field *E* exceeds *E*_*c*_. A stronger electric field will create a larger area of electroporated tissue (Eq 6; Fig 10B), which will be more permeable and hence more susceptible to LVPs.

Because of the limit of the critical electric field, LVPs cannot generate the sufficient electroporation area to achieve ablation. However, HVPs exceed the electroporation threshold (*U*_*m*_>*U*_*c*_), which substantially enhances the electroporated area of the cells (Eq 5) and tissues (Eq 6). Therefore, before LVPs are applied, HVPs can produce a large electroporated area and increase the susceptibility of the area to LVPs.

Although HVPs create suitable conditions for tissue ablation, LVPs ablate more effectively. The type of electroporation that occurs is controlled by the size of the pores in the cell membrane. If the pores are sufficiently small to automatically recover upon removal of the pulse, reversible electroporation occurs. In contrast, once the pores become too large to recover after the pulse, IRE occurs.

**Fig 11:**
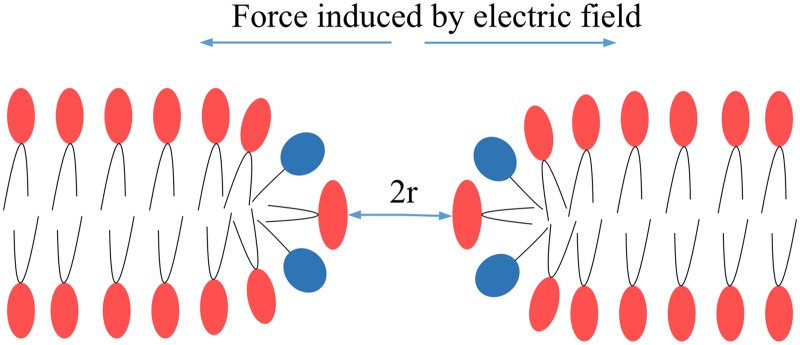
Schematic demonstrating that the force induced by the electric field contributes to the development of electroporation; *r* is the pore radius.

The pore dynamics are controlled by the force induced by the electric field applied to the lipid molecules (Fig 11). Three types of forces have been identified in the electroporation process [44], which are summed as follows:

$$F_{E}=\rho E+\frac{1}{2}E^{2}\nabla \varepsilon +f$$ (7)

where *ρ* is the space charge, ∇ε is the gradient of the permittivity, and *f* is associated with the electrostrictive strain. According to the conservation of momentum,

$$F_{E}t=mv$$ (8)

where *m* is the mass of a lipid molecule, *v* is the velocity of a lipid molecule, and *t* is the pulse time when *F*_*E*_ acts on a lipid molecule.

The displacement *s* of a lipid molecule is equal to the pore diameter, and the size of the pore is generally evaluated by the pore radius. Thus, the pore radius *r* can be obtained from the equation below:

$$r=\frac{s}{2}=\frac{\int_{0}^{t}vdt}{2}=\frac{\rho Et^{2}}{4m}+\frac{E^{2}t^{2}\nabla \varepsilon }{8m}+\frac{ft^{2}}{4m}$$ (9)

Eq 9 shows that the pore size is related to the electric field strength and pulse width. Although the electric field influences pore development, the pulse width plays a more important role [45]. For example, as shown in Table 6, the three coefficients of HVPs (4 kV/cm) are always less than those of LVPs (0.4–1 kV/cm) in Eq 9, even at higher HVP doses (dose: 32 kV^2^μs/cm^2^ at 4 kV/cm) than LVP doses (dose: 16 kV^2^μs/cm^2^ at 0.4 kV/cm). Particularly, for the first term, the coefficient for the LVPs is three orders of magnitude greater than that of the HVP. Therefore, the LVP contributes more to pore development and increases the likelihood of IRE. Even if the HVP produces a large electroporated area, it contributes less to pore development; hence, the ablated region would be small. In contrast, the LVP produces a small electroporated area but has a great influence on pore size. The SHLVP protocol takes advantage of both these processes; the HVP has a stronger electric field that creates a larger electroporated area, making the electroporated area more susceptible to the LVP and resulting in highly efficient necrosis.

**Table 6:** Pore sizes resulting from electric force by different pulsation protocols.

Protocol	*E* (kV/cm)	*t* (μs)	Dose (kV^2^μs/cm^2^)	Coefficient in Eq 9		
				First term	Second term	Third term
**HVP**	4	2	32	4×10^−7^	0.08	10^−12^
**LVP**	0.4	100	16	1×10^−4^	2	2.5×10^−9^
**LVP**	0.6	100	36	1.5×10^−4^	4.5	2.5×10^−9^
**LVP**	0.8	100	64	2×10^−4^	8	2.5×10^−9^
**LVP**	1	100	100	2.5×10^−4^	12.5	2.5×10^−9^

The mechanisms by which cell death occurs when electric pulses are applied continue to be actively investigated. One mechanism with experimental support posits that the inability of large pores to recover could lead to permanent cell membrane disruption and cell death [46,47]. The above theoretical analysis indicates that SHLVPs can easily create large pores in the cell membrane via HVPs and LVPs, resulting in cell death. However, biological mechanisms could also contribute and may eventually result in cytotoxic effects. As introduced by Pakhomov et al., delayed electrosensitization occurs when the first pulse train renders cells more sensitive to the cytotoxic effect of the second pulse train, as applied at certain time intervals, thereby increasing the efficiency of the entire treatment [48,49]. For SHLVPs (HVPs+LVPs), since HVPs increase membrane permeabilization because of the higher electric field (Eq 5), they may be more efficient at engaging electrosensitization. Davalos also hypothesized that membrane permeabilization-induced osmotic imbalances, which flush ions in and out of cells, may eventually result in cell death after IRE [50]. Pores in the cell membrane serve as ion transport channels upon the application of electric pulses. Electropermeabilized membranes are conductive for Na+, K+, and Cl− ions. As such, electropermeabilized cells may not maintain the normal transmembrane concentration gradients of these ions, which are key components of the net cellular osmotic balance, and osmotic imbalances can result in cell death [6,51]. For SHLVPs (HVPs+LVPs), HVPs can increase the area of membrane permeabilization because of the stronger electric field (Eq 5), then larger areas of membrane permeabilization provide broad channels through which ions can be transported into or out of cells by LVPs, leading to osmotic imbalances that result in cell death. However, none of these mechanisms has been experimentally modeled as a cause of the synergy of SHLVPs. Further experimental validation will be investigated for the synergistic mechanisms.

## Conclusion

In this study, different electric pulse protocols were applied to potato tuber slices. The results confirmed that a combination of HVPs and LVPs (i.e., SHLVPs) effectively ablated tissue. The SHLVP protocol employed HVPs and LVPs in a synergistic manner to produce more potent necrosis than either of these protocols alone, even when the SHLVP dose was similar to or lower than the HVP or LVP doses. However, the application of LVPs+HVPs could not increase the ablation area. Real-time temperature monitoring confirmed that the protocols used in this study resulted in non-thermal ablation. The calculations presented in the discussion provide theoretical support for the observed synergistic effects. Finally, an optimized combination of LVPs and HVPs may provide therapeutic benefits, which will be the subject of future work.
